# Standing on Principle: The Global Push for Environmental Justice

**DOI:** 10.1289/ehp.115-a500

**Published:** 2007-10

**Authors:** Luz Claudio

Climate change, acid rain, depletion of the ozone layer, species extinction—all of these issues point to one thing: environmental health is a global issue that concerns all nations of the world. Now add environmental justice to the list. From South Bronx to Soweto, from Penang to El Paso, communities all over the world are finding commonality in their experiences and goals in seeking environmental justice.

Environmental justice was defined by Robert Bullard, director of the Environmental Justice Resource Center at Clark Atlanta University, in his seminal 1990 work *Dumping in Dixie: Race, Class, and Environmental Quality* as “the principle that all people and communities are entitled to equal protection of environmental and public health laws and regulations.” In countries around the world, the concept of environmental justice can apply to communities where those at a perceived disadvantage—whether due to their race, ethnicity, socioeconomic status, immigration status, lack of land ownership, geographic isolation, formal education, occupational characteristics, political power, gender, or other characteristics—puts them at disproportionate risk for being exposed to environmental hazards. At a global scale, environmental justice can also be applied to scenarios such as industrialized countries exporting their wastes to developing nations.

In either case, “environmental and human rights have no boundaries, because pollution has no boundaries,” says Heeten Kalan, senior program officer of the Global Environmental Health and Justice Fund of the New World Foundation in New York City. “Environmental justice organizations are starting to understand that they are working in a global context.”

## Global Awareness

The history of international efforts in environmental justice parallels the series of agreements and conventions held around the globe to address environmental issues. Bullard recounts that during the 1992 Earth Summit in Rio de Janeiro, Brazil, there was not much official discussion about environmental justice in the context of human health. “Most of the official discussion centered around saving the Amazon and other ecosystems. Human health and urban centers were not considered part of the ‘environment,’” he says.

However, Bullard and other U.S. environmental justice leaders had already met in Washington, DC, at the First National People of Color Environmental Leadership Summit a year earlier, where they drafted the *Principles of Environmental Justice*, a document to guide grassroots organizing. “When we went to Rio in 1992 we found that some groups had translated the *Principles* into Portuguese and were circulating the document to local community leaders at the summit,” remembers Bullard.

Ten years later, during the World Summit on Sustainable Development held in Johannesburg, South Africa, the issue of environmental inequity was formally recognized by the leadership of the summit. “By the time we went to Johannesburg, environmental justice had really caught on across borders as part of the whole idea of sustainable development,” says Bullard. Just two years earlier, the eight UN Millennium Development Goals that resulted from the UN Millennium Summit held in New York City had encompassed environmental sustainability as a goal that would require a reduction in inequality.

International organization around environmental justice issues takes several different forms. Broad networks of community-based organizations can work on different issues affecting the disenfranchised and come together on matters related to the environment. Other groups may organize a particular labor sector to improve worker health. On an international scale, community-based groups in different countries who find themselves fighting similar environmental problems can unite in order to synergize their efforts.

“The issue of globalization is one of common concern to the environmental justice movement in many developing countries,” says Michelle DePass, program officer of the Environmental Justice and Healthy Communities Program at the Ford Foundation. Concerns about globalization can bring together a wide range of stakeholders including workers, academics, and community leaders for whom increased industrial development is a common denominator.

## Into Action

The Brazilian Network on Environmental Justice is an example of how groups can come together to address common concerns. This network brings together about 100 varied organizations including unions, academic centers, associations, ethics groups, community-based organizations of indigenous peoples, and descendants of enslaved Africans brought to Brazil, all with the common goal of improving the conditions for vulnerable populations in that nation.

Utilizing the *Principles of Environmental Justice*, the Brazilian network serves as a forum for debate, strategic planning, and mobilization by organizations and affected populations. Network meetings include members from other South American countries with common interests.

Marcelo Firpo, a network organizer and senior researcher at the Oswaldo Cruz Foundation in Rio de Janeiro, sees that what unites these varied organizations is their concern for issues of human rights and the effects of globalization on health and the environment. He offers the example of Petrobras, a Brazilian oil company that has become a major player in the global market. Because the current government in Brazil does not permit oil exploration in the Amazonian native reservations, Petrobras has begun exploration in Ecuador, where there are no such restrictions. “This kind of situation necessitates international collaboration,” says Firpo.

Throughout the world, disadvantaged communities typically suffer the highest burdens of environmental degradation. One group that is often threatened by environmental hazards in developed and developing countries alike is rural farmworkers. These workers often suffer from the effects of disproportionate exposure to pesticides and other chemical agents as well as lack of access to health and education services, among other hindrances.

In Brazil, for example, 10% of the urban population over 5 years of age is illiterate whereas in the rural population this rate is as high as 30%, according to Frederico Peres, a researcher at the Center for Workers’ Health and Human Ecology at the Oswaldo Cruz Foundation. So workers often cannot understand the written technical information about pesticides provided by chemical manufacturers. Protective gear is often ineffective or nonexistent, and government protections regulating use and disposal of pesticides may not be consistently applied to these vulnerable populations.

Peres has mobilized farmworkers and created educational materials on the safe use of pesticides that do not require literacy to be understood by the workers. In conducting this work, Peres connected with similar organizations in Mexico, Chile, Ecuador, Panama, and Argentina and observed that comparable situations take place in these countries. “The problems are the same: illiteracy, lack of government support, the strong influence of chemical industries to promote pesticide use—all of these are the same throughout Latin America,” says Peres.

Farmworkers in South Africa face similar situations as those in Brazil. Labor conditions on South African farms are among the poorest of all employment sectors in that country, and until recently farm work was effectively unregulated. Similar to Brazil for Latin America, South Africa is the largest importer of pesticides in sub-Saharan Africa, so pesticide exposure is a significant hazard for South African farmworkers. Leslie London, a professor of public health at the University of Cape Town, has collaborated with South African farmworkers for many years to address their environmental justice concerns. But as he noted in the January/March 2003 issue of the *International Journal of Occupational and Environmental Health*, “The legacy of apartheid for the health and the dignity of farm workers has proved to be so deep-rooted that efforts towards redress in the new democracy have had only limited success. . . . [I]t is the underlying powerlessness of farm workers that is both at the root of violations of farm workers’ human rights and also responsible for the substantial burden of mortality and morbidity suffered by farm workers and their families.”

## Going International

Upon interacting with each other, some organizations in the environmental justice movement across the globe are discovering that although each case has its own particular circumstances, there are many common experiences that can inform each other’s struggles for environmental justice. For example, members of the Farmworker Association of Florida have been exchanging visits with citrus farmers in Brazil to trade ideas on how to address environmental justice issues. They found that some of their local circumstances were different, primarily the fact that in the United States most of the farmworkers are immigrants, whereas in Brazil they are mostly nationals. “This makes a huge difference since in Brazil [workers] have the right to unionize to seek better working conditions,” says Tirso Moreno, general coordinator of the Farmworker Association of Florida.

Yet, during these exchanges, the workers from both countries discovered that they had been facing similar working conditions established by the same multinational agrobusiness companies. “Some of the information that we had [was of use to] the Brazilians and vice versa because many of these multinational companies are the same ones with different names,” says Moreno. “That is why there is a lot more interest in collaborating internationally. While the details may be different in each country, the struggles are the same.”

Organizations like Via Campesina, an international organization of small and medium-sized agricultural producers based in Indonesia with members in 56 countries, aim to organize farm workers throughout the world who are affected by similar issues. Jose Adilson de Medeiros, president of the São Lourenço [Brazil] Rural Producers Association, says of these groups, “If [other environmental justice groups] know how to solve a problem, they can tell us how they did it. We learn from each other’s mistakes so we don’t have to make a mistake again to get there.”

Another issue-based environmental justice network is the Global Alliance for Incinerator Alternatives (GAIA). This organization, headquartered in the Philippines, aims to coordinate efforts to reduce waste and stop incineration around the world with a particular focus on representing disadvantaged communities in both developed and developing countries.

With members from 77 countries and expanding, GAIA can mobilize quickly and globally to take coordinated actions. Its approach includes sharing information electronically, coordinating regional meetings, developing joint strategies for community organizing, and hosting international training sessions where skills can be shared. One effective strategy the group has used is letter-writing campaigns that include signatories representing organizations from many countries. GAIA is current mobilizing Asian members in opposition to an effort by the Japanese government to enter into bilateral agreements allowing the export of waste for burning in less-developed countries in the region.

Another approach taken by the environmental justice movement is to address the international bodies that support projects that may affect disadvantaged populations. For example, GAIA has launched a campaign to stop the World Bank from funding incinerators around the world. To achieve this goal, GAIA locates expert researchers who can share needed information on the health effects of incineration with members near the proposed incinerator where the information may not be readily available. They also facilitate linkages between members who may be campaigning against similar technologies or against the same incinerator vendor. In this way, environmental justice organizations can share strategies and information quickly and effectively.

The flow of information is highly bidirectional in the international environmental justice movement, providing models for both North-to-South as well as South-to-North exchange. For example, community-based organizations in the Philippines, where the government passed a national ban on incineration in 1999, are able to share with others around the world how they were able to achieve this in their country. And in Kenya, lawyers are required to train in environmental law through continuing education programs such as those managed by the Institute for Law and Environmental Governance (ILEG). “In the United States, we can learn a lot from organizations like ILEG,” says DePass, who is herself an environmental lawyer who will be leading a delegation of U.S. lawyers to visit ILEG for consultation on environmental justice strategies.

## A Common Cause

Increasingly, due to globalization and the advance of multinational corporations, communities around the world find they are fighting the same battles. One such example began in Diamond, a black community in Norco, Louisiana, which is home to 130 petrochemical facilities, incinerators, and landfills in what is known by some as the Chemical Corridor and by others as Cancer Alley. There, a local school teacher named Margie Richard and other neighbors founded Concerned Citizens of Norco in 1990 and began demanding that Shell Corporation, the owner of the nearby petrochemical facilities, take responsibility for its pollution by relocating affected residents to a cleaner area.

To achieve this, the group engaged in highly visible campaigns at the state, national, and international levels, culminating with Richard’s presentation in 2001 at the international headquarters of Royal/Dutch Shell in the Netherlands. Shell agreed to relocate those in the community who wished to leave the area and to reduce its emissions by 30%. This unprecedented victory won Richard the 2004 Goldman Environmental Prize (considered the Nobel Prize for environmental activism). With this increased visibility and recognition, Richard began traveling abroad to talk about the environmental justice movement and likening this experience to the wider issue of international human rights.

Communities in other parts of the world are now utilizing tactics similar to those used by Concerned Citizens of Norco. For example, Desmond D’Sa, a resident of South Durban, South Africa, and chairperson of the South Durban Community Environmental Alliance, has engaged the leadership of Shell Corporation directly to deal with environmental issues similar to those in Norco. Other communities in Texas, the Philippines, Nigeria, Brazil, Curaçao, and Russia have brought similar complaints to Shell’s annual General Meetings.

Friends of the Earth International, described as the world’s largest grassroots environmental network with 70 national member groups and approximately 5,000 local activist groups, serves as an umbrella organization under which many of the communities organizing for environmental justice can find common ground for action. In a 2003 report titled *Behind the Shine*, Tony Juniper, executive director of Friends of the Earth in the UK, states that shareholders and investors in large corporations have rights established in law through which they can hold companies accountable; however, this cannot be said for the people who live next door to polluting facilities. Joining forces therefore helps these communities have their voices heard at the corporate table.

In recent months, attention has been focused on environmental justice issues within Europe, where poor and ethnically marginalized peoples in Central and Eastern Europe often face harsh environmental health conditions. “With the recent enlargement of the European Union to include countries of Central and Eastern Europe, the need for environmental justice across a more stratified society, especially as it relates to the promotion of human health, is increasingly evident,” says Diana Smith, director of communications at the Health and Environment Alliance (HEAL), headquartered in Brussels. The alliance mainly addresses environmental justice within the context of the 1998 Aarhus Convention, which specifically links environmental rights and human rights.

HEAL and its member organization, the Centre for Environmental Policy and Law, produced the groundbreaking August 2007 report *Making the Case for Environmental Justice in Central and Eastern Europe* to raise awareness and advocate policy action against the deleterious environmental and human health conditions of poor and otherwise marginalized groups in Central and Eastern Europe. The report cites the case of a displaced persons camp sited near a mine complex in Northern Mitrovica, Kosovo. A 2005 WHO study visit to the camp showed that 88% of the children aged 6 years and younger had lead poisoning severe enough to require immediate medical intervention.

The global push for environmental justice can only be expected to grow—and the time for action is ripe. As Bullard summarizes, “if you live on the wrong side of the tracks and you are denied a good environment, then you need environmental justice. It is the same struggle everywhere.”

## Figures and Tables

**Figure:**
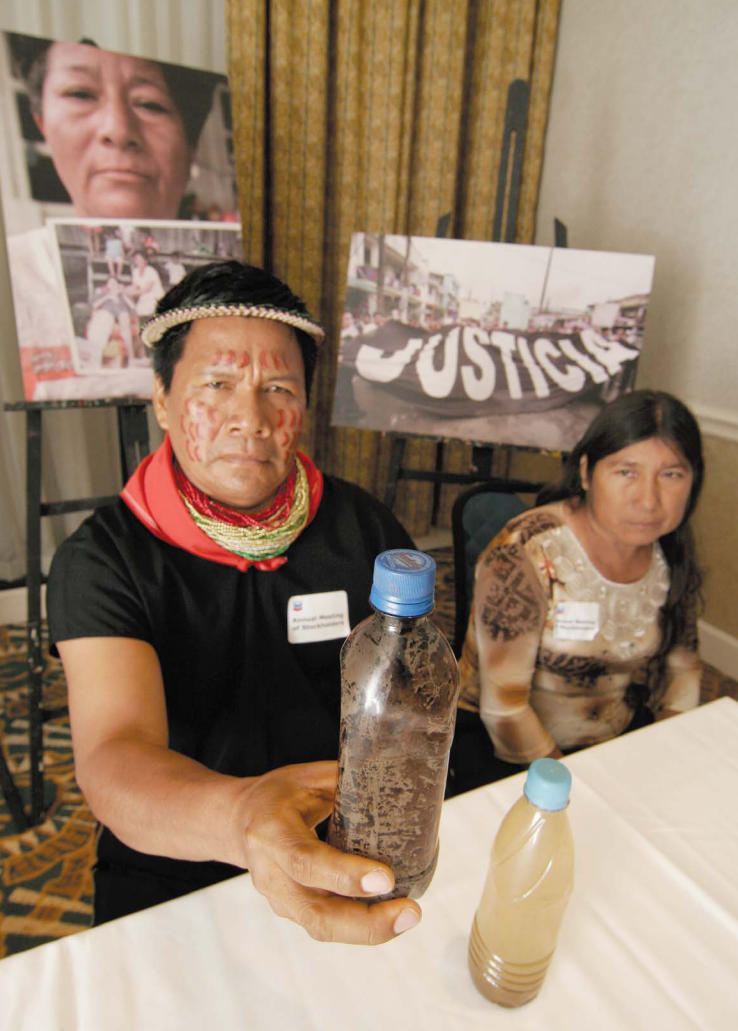
Emergildo Criollo Quenema (left), Cofán indigenous leader from Ecuador, and resident Rita Maldonado (right), brought bottles of polluted water to a 2006 ChevronTexaco shareholders’ meeting in Houston, Texas. In a class-action lawsuit launched by more than 30,000 Ecuadorians, ChevronTexaco is accused of dumping 18 billion gallons of toxic water into the rainforest where the Cofán live. A groundswell of actions such as this is bringing the voices of disadvantaged populations to the corporate table.

